# Within-Mice Comparison of Microdialysis and Fiber Photometry-Recorded Dopamine Biosensor during Amphetamine Response

**DOI:** 10.1021/acschemneuro.2c00817

**Published:** 2023-04-12

**Authors:** Aske L. Ejdrup, Joel Wellbourne-Wood, Jakob K. Dreyer, Nina Guldhammer, Matthew D. Lycas, Ulrik Gether, Benjamin J. Hall, Gunnar Sørensen

**Affiliations:** † Department of Circuit Biology, H. Lundbeck A/S, Valby 2500, Denmark; ‡ Department of Bioinformatics, H. Lundbeck A/S, Valby 2500, Denmark; § Department of Neuroscience, Faculty of Health and Medical Sciences, Maersk Tower 7.5, University of Copenhagen, Copenhagen 2200, Denmark

**Keywords:** Fiber photometry, microdialysis, biosensors, dopamine, striatum, amphetamine

## Abstract

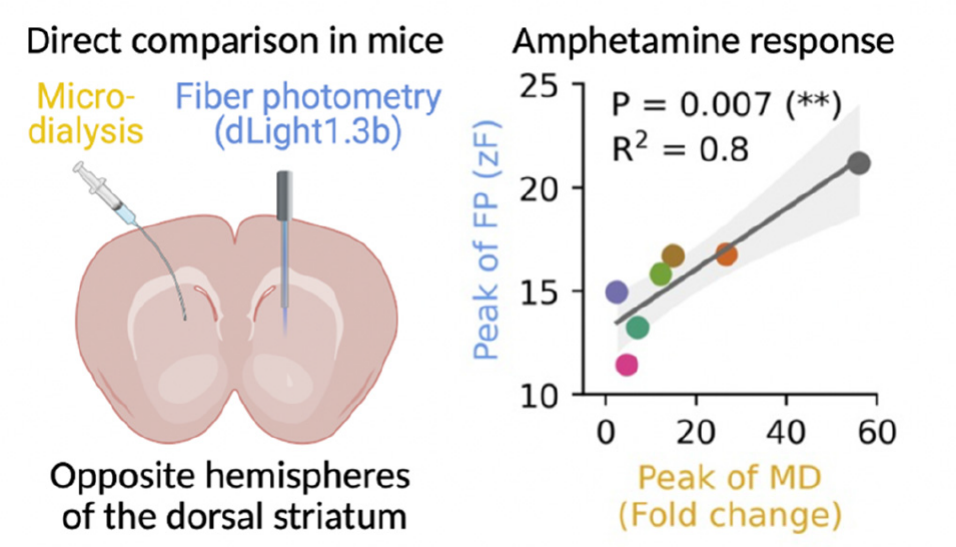

A fundamental concept in neuroscience is the transmission of information between neurons via neurotransmitters, -modulators, and -peptides. For the past decades, the gold standard for measuring neurochemicals in awake animals has been microdialysis (MD). The emergence of genetically encoded fluorescence-based biosensors, as well as *in vivo* optical techniques such as fiber photometry (FP), has introduced technologically distinct means of measuring neurotransmission. To directly compare MD and FP, we performed concurrent within-animal recordings of extracellular dopamine (DA) in the dorsal striatum (DS) before and after administration of amphetamine in awake, freely behaving mice expressing the dopamine sensor dLight1.3b. We show that despite temporal differences, MD- and FP-based readouts of DA correlate well within mice. Down-sampling of FP data showed temporal correlation to MD data, with less variance observed using FP. We also present evidence that DA fluctuations periodically reach low levels, and naïve animals have rapid, predrug DA dynamics measured with FP that correlate to the subsequent pharmacodynamics of amphetamine as measured with MD and FP.

## Introduction

Monitoring the concentration of extracellular signaling molecules is important to understand brain function and pathology. Several methods with differences in selectivity, resolution, and duration have been used to investigate the characteristics of neuronal signaling. Electrochemical methods such as fast-scan cyclic voltammetry (FSCV) can achieve subsecond temporal resolution, but chemometric data processing restricts the total duration of the measurement to a minute for most practical purposes. FCSV offers some molecular specificity but requires the molecule to be electroactive and free from interference from other molecules with similar electrochemical properties.^1,2^ This limits the technique to brain regions with high neurotransmitter specificity, such as dopamine (DA) in the striatum. In comparison, microdialysis (MD) provides a lower sampling rate in the range of many minutes to hours,^3^ although faster rates have been described.^4^ An advantage of MD, however, is its high specificity for molecular species and their metabolites via analysis of dialysates using high performance liquid chromatography (HPLC) or mass spectrometry (MS).

Genetically encoded biosensors offer an optical method for monitoring neurotransmission *in vivo* at a higher time resolution than MD and at lower analyte concentrations than FSCV.^5−8^ The fluorescent signal from these biosensors is typically sampled at a time resolution comparable to or higher than FSCV^9^ and offers the ability to capture changes in neurotransmitter levels over longer time scales. Biosensors for a host of molecules have been developed, making it a versatile and popular strategy for monitoring *in vivo* changes in neuronal signaling.^10,11^ A weakness of data from biosensors, however, is its relative nature. Whereas MD readily provides measures of concentration, through analysis of dialysates using HPLC or MS, and FCSV can be calibrated *ex vivo* to provide changes in concentration, biosensors provide relative changes in transmitter concentration. In fact, many FP experiments report changes in z-scored fluorescence levels relative to a fluctuating baseline.^12,13^ While this enables easy and reproducible analysis, biological information is lost in z-scoring and individual differences in baseline might be influencing the interpretation of subsequent sections of data.

The aim of this study was to directly compare extended FP recordings of the dopamine (DA) biosensor dLight1.3b fluorescence to DA measurements derived via MD and analyzed using HPLC. We sought to use the selectivity and calibrated nature of MD recordings to enhance our understanding of DA neurotransmission as observed through FP. Further, we investigated if this information could provide new insights into DA neurotransmission in response to administration of amphetamine. Our results, obtained from the dorsal striatum of both hemispheres of single mice, showed that both the time course and peak of amphetamine-induced DA release correlate well between MD and area under curve (AUC) of the FP time series. Notably, FP data showed lower within-group variance. Using the correlation established between the two methods from the amphetamine administration, we infer that at baseline striatal DA frequently reached levels below the detection-limit of our FP setup, if only for subsecond intervals. Lastly, our analysis of the FP signal showed that peak DA release rates, preinjection, correlate with the magnitude of response induced by amphetamine as measured by both MD and FP, highlighting the power of insights into rapid temporal dynamics of extracellular neurotransmitter concentrations.

## Results

### Concurrent MD and FP Recordings

**Figure 1:**
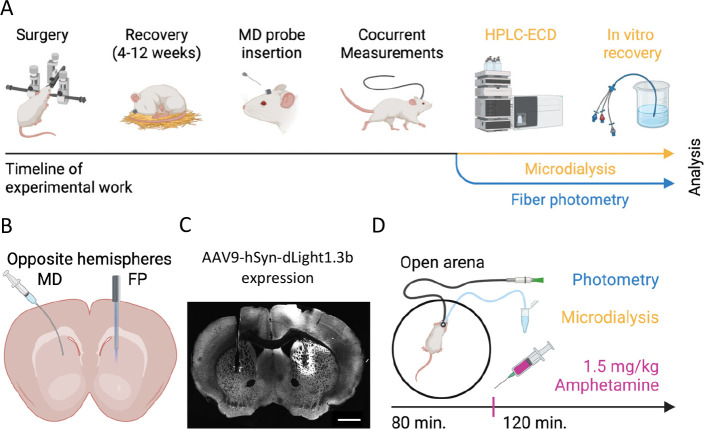
Concurrent within-mouse microdialysis and fiber photometry recordings. (A) Schematic for experimental setup. Mice undergo surgery for both MD and FP measurements. Three hours before concurrent recordings an MD probe was inserted. MD dialysate was analyzed using HPLC, and baseline values were corrected following *in vitro* recovery. FP data can be analyzed immediately after acquisition. (B) Sketch of recording location paradigm. Hemisphere placement of MD cannulas and FP lenses was switched between mice. (C) Representative immunohistochemical image of a coronal brain slice of the mouse striatum showing guide canula and probe placement, left, and endogenous cpGFP fluorescence from dLight 1.3b and lens placement, right. (D) Schematic for the measurement sessions. Mice were placed in a circular open arena for 3 h of habituation after which 80 min of baseline data was sampled prior to injection of 1.5 mg/kg amphetamine. Following injections another 120 min were sampled. MD and FP were sampled simultaneously throughout the experiment.

To directly compare measurements of extracellular DA between MD and FP, we performed concurrent recordings in the dorsal striatum of opposing hemispheres in the same mice (Figure 1A–C). We achieved this by implanting a MD guide cannula containing a dummy probe in one hemisphere. In the opposite hemisphere of the same animal, we injected an adeno-associated virus (AAV) encoding dLight1.3b^5^ expressed under control of the human synapsin promoter (AAV9-hSyn-dLight1.3b) along with implantation of a 200 μm optical fiber for fluorescent signal recording followed by 3–4 weeks of recovery. On the day of the experiment, we exchanged the dummy probe for an MD probe protruding an additional 1 mm from the guide cannula into the tissue and attached the optical fiber to the FP lens. Mice were habituated for 3 h in a circular open arena of 14.5 cm in diameter before initiation of recordings. We sampled concurrently with both techniques for an 80 min baseline period, then injected the mice with 1.5 mg/kg amphetamine subcutaneously (sc). We then placed them back in the arena for 120 min (Figure 1D). Data analysis was limited to 60 min prior to injection due to initial photobleaching. Amphetamine was chosen as a circuit activator because of its well-documented effect on extracellular DA in dorsal striatum of rodents as result of its high affinity interaction with the dopamine transporter (DAT).^14,15^ Following the in-life phase of the experiments, the MD samples were analyzed on HPLC with a lower limit of detection of 0.25 nM. *In vitro* recovery of DA across the MD probe membrane was determined in a separate group to be 8.3% ± 1.1. From this we inferred a baseline value of 12.9 nM ± 1.8 for the saline group and 10.5 nM ± 1.4 for the amphetamine group for a combined 11.8 nM ± 1.2 across the cohorts. These values are similar to those from previous literature reports.^16^ Lastly, fiber photometry signals were preprocessed and converted to z-scored d*F*/*F*_0_ (zF) for cross-animal comparison (Figure S1A–C and methods).

### FP Shows Earlier Peak DA and Low Variation across Animals in Response to Amphetamine Challenge

**Figure 2:**
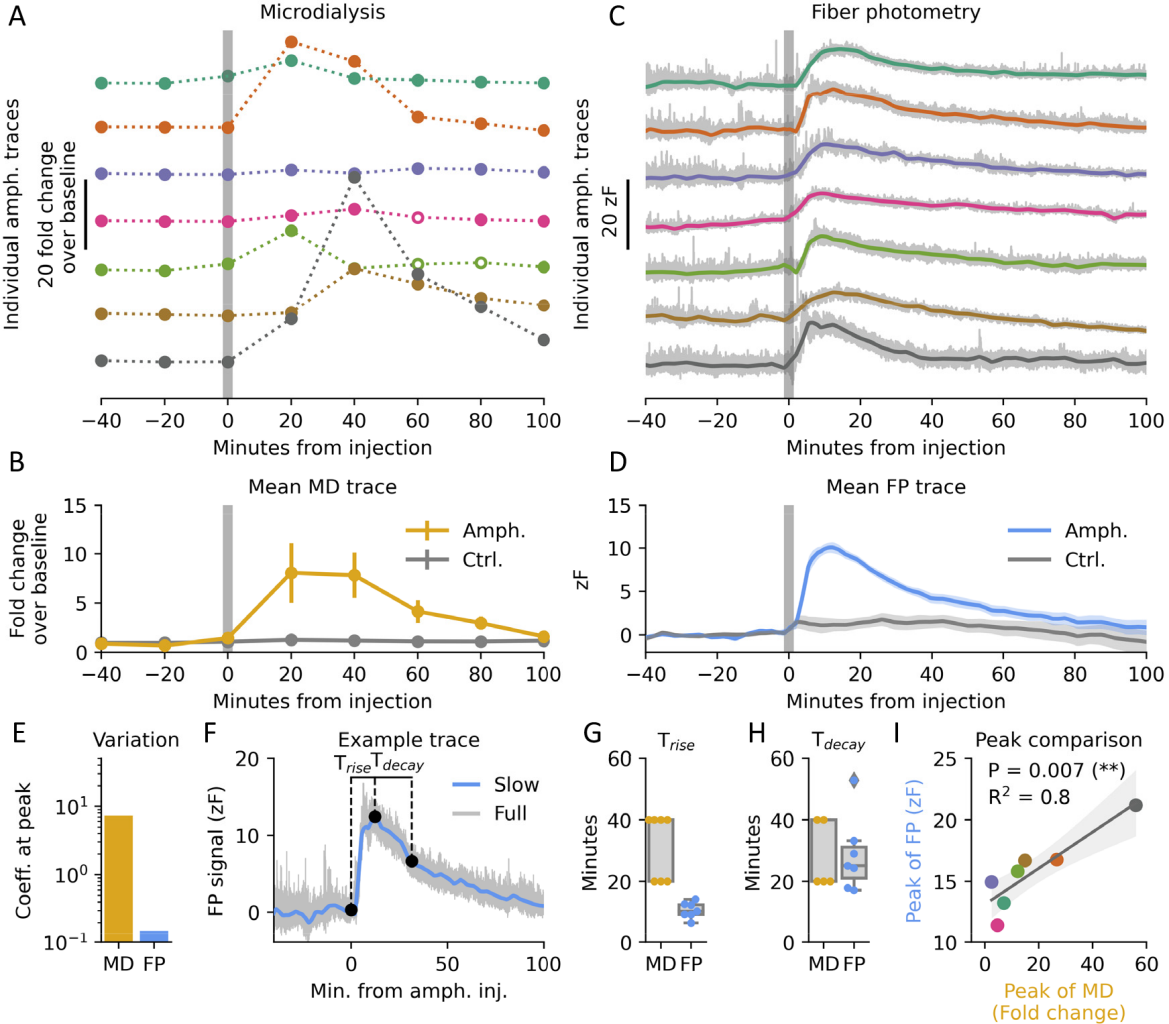
Comparison of amphetamine response. (A) MD traces presented as fold change from baseline for all seven amphetamine-injected mice. Hollow points indicate samples that did not pass the signal-to-noise ratio threshold during HPLC analysis of dialysates. (B) Mean MD trace for amphetamine and saline-injected mice. Error bars indicate SEM. (C) Individual FP traces representing dLight1.3b fluorescence for amphetamine-injected mice plotted as z-scored d*F*/*F*_0_ (zF). Thick, colored lines represent a 0.02 Hz lowpass-filtered signal, with gray background traces showing the unfiltered signal. Colors matching to (A) indicates concurrent recording from same mouse. (D) Mean trace of dLight1.3b fluorescence low-pass filtered at 0.02 Hz for amphetamine and saline-injected mice. Shaded areas indicate SEM. (E) Coefficient variation at peak response to amphetamine for MD and FP. (F) Representative FP trace of dLight1.3b fluorescence low-pass-filtered at 0.02 Hz showing quantification of rise time (*T*_rise_) and decay (T_decay_) of amphetamine response. Unfiltered trace shown as gray background. (G) Rise time of amphetamine response as measured by MD and FP. (H) Half-life of amphetamine response as measured by MD and FP. (I) Peak amphetamine response in MD response correlated to FP dLight1.3b fluorescence. Shaded area indicates 95% C.I. Two-sided linear regression, *P* = 0.007, *R*^2^ = 0.8, *n* = 7. Colors match to traces in (A) and (C).

We first investigated the overall changes in extracellular DA in response to amphetamine as observed with MD. Figure 2A shows DA levels relative to baseline for each mouse with samples offset for visibility. The empty circles indicate samples that did not meet technical thresholds for confident peak detection during HPLC and were not included in the remaining analyses. We found that amphetamine administration led to an increase in concentration of DA in the dialysate of all animals. The variation between animals was large, however, with a 2-fold increase of DA relative to baseline at the lowest (Figure 2A, purple) and a nearly 60-fold increase at the highest (Figure 2A, gray). DA in individual mice peaked in dialysate collected either at minute 0 to 20 or minute 20 to 40 after amphetamine injection, with a peak average concentration of 8.07 (± 3.03)-fold relative to baseline at minute 0–20 (Figure 2B). Importantly, the peak amphetamine response did not correlate to wait time between surgery and recording (Figure S1D). When converted to absolute concentrations, amphetamine-exposed animals reached 95.3 nM ± 35.8 (Figure S1E). Overall, our MD results are in accordance with similar experiments reported in literature.^17−20^

We then investigated the concurrent fluorescence response as measured by FP. Figure 2C shows offset response z-scored to preinjection baseline. We again found a consistent increase in fluorescence after amphetamine, as FP showed a response of 12–21 standard deviations above normalized baseline across all animals. Peak fluorescence occurred from 7 to 14 min after administration, and the average fluorescence trace peaked after 11 min at 10.05 ± 0.56 standard deviations from baseline (Figure 2D). When quantifying variation at peak, we found FP had a coefficient of variation 1.5 orders of magnitude lower than MD (Figure 2E). Furthermore, the power spectral density of the FP signal changed markedly after amphetamine, where slower oscillations dominated the signal (Figure S1F–I).

The subsecond temporal resolution of FP allowed us to investigate the kinetics of the response to amphetamine in finer detail than for MD. We defined rise time (*T*_rise_) as the time from administration to peak of the low pass filtered signal, and decay time (*T*_decay_) as the time from peak to e^–1^ (∼37%) of peak fluorescence (Figure 2F). These values are poorly determined in MD without advanced interpolative modeling due to the coarse sampling rate. In contrast, they are easily quantified with high precision in FP. For MD, *T*_rise_ was between 0–20 and 20–40 min, whereas FP showed a consistent 10.6 ± 1.0 min (Figure 2G). Similarly, MD showed a *T*_decay_ between 20 and 40 min, whereas FP had a larger variation than its rise-time, with a decay at 28.5 ± 4.6 min (Figure 2H). We observed no correlation between *T*_rise_ and *T*_decay_ for FP (Figure S1J). Finally, despite significant methodological differences, we observed a strong correlation of peak amphetamine response between MD and FP, suggesting shared biological information (Figure 2I).

Taken together, we demonstrate a methodology difference in the rise time and the variance of DA response at peak amphetamine effect. No differences between MD and FP were observed for decay time or peak effect size following amphetamine challenge.

### High Translatability between MD and FP Amphetamine Response

**Figure 3:**
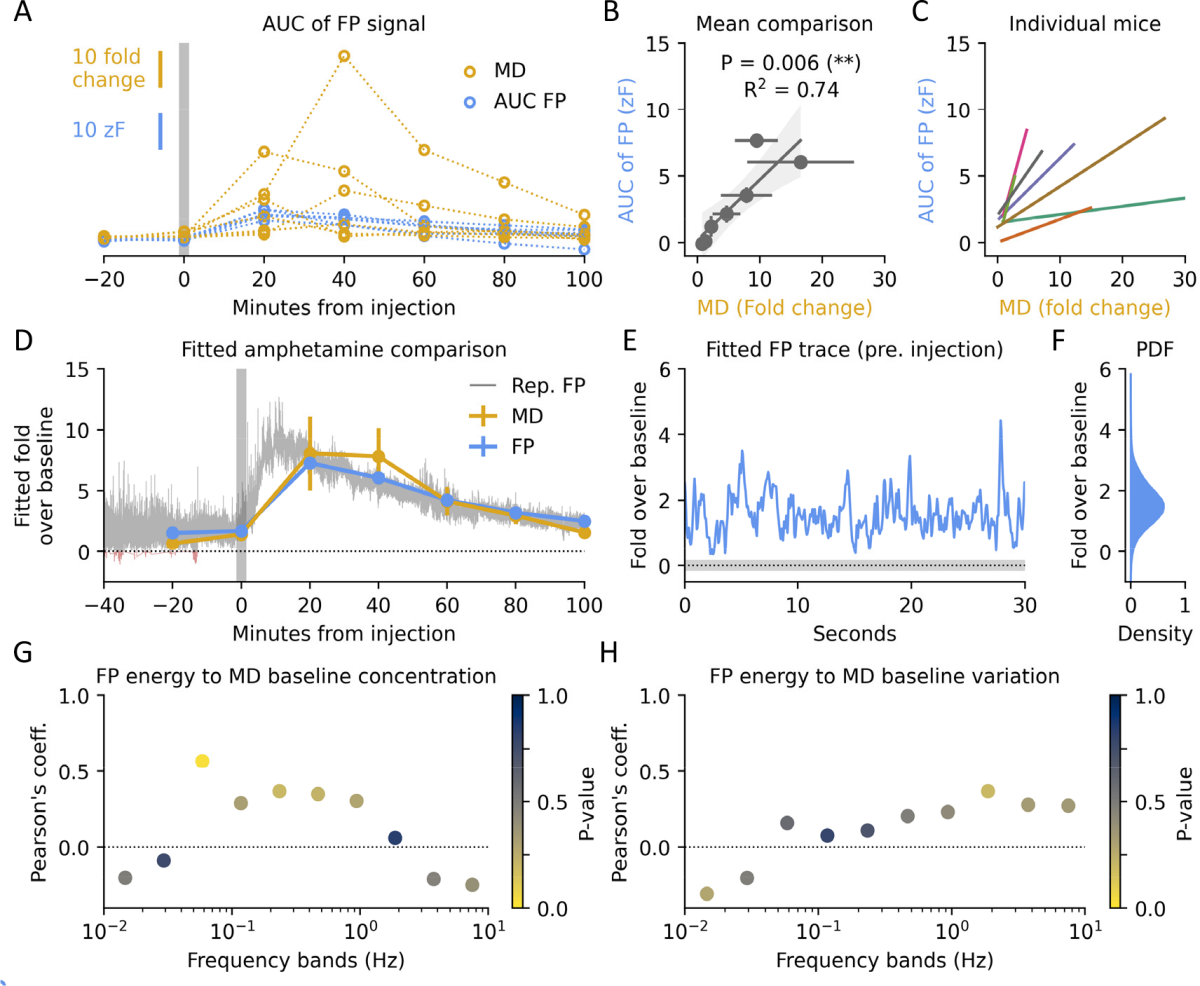
Temporal correlation and baseline estimation. (A) Individual MD traces plotted alongside down sampled FP dLight1.3b fluorescence traces by calculating area under the curve of the preceding 20 min. (B) Correlation between mean values of MD and down sampled FP. Error bars indicate SEM for the individual time points. Shaded area indicates 95% C.I. of the regression. Two-sided linear regression, *P* = 0.006, *R*^2^ = 0.74, *n* = 7. (C) Individual regression lines of transformed FP and MD for each mouse. Colors match traces in Figure 2A,C. Individual statistics and data points can be found in Figure S2A. (D) Mean of down sampled FP during amphetamine injection fitted to MD by linear regression as in (B). Background trace is a representative, fitted, unfiltered FP trace. Error bars indicate SEM. (E) Representative fitted FP trace before injection. Shaded area indicates 95% C.I. of intercept in (B). (F) Probability density function (PDF) of FP values across all mice after application of fit in (B). Only preinjection data are included. (G) Pearson’s coefficient between MD baseline values and relative energy at different frequency bands in FP traces. Color indicates statistical significance. No values were significant (α = 0.05) after Bonferroni correction for multiple testing. (H) Pearson’s coefficient between MD baseline variation and relative energy at different frequency bands in FP traces. Color indicates statistical significance. No values were significant (α = 0.05) after Bonferroni correction for multiple testing.

So far, we have compared MD and FP with little consideration for what underlying DA signal they reflect. MD is seemingly used to capture tonic levels of DA,^21^ but few studies clearly define what tonic DA is.^22^ Given MD is the most established technique and frequently used to investigate disease states, drugs of abuse, and pharmaceutical interventions,^23^ we wanted to understand what underlying phenomena are being measured. To that end, we hypothesized that DA measurements obtained from MD reflect AUC of all DA fluctuations recorded with FP, plus an offset equivalent to the minimum DA levels reached. In other words, MD captures phasic DA as well as any potential minimum basal levels. To approach a direct comparison, we down-sampled our FP signal to the same rate as our MD measurement by taking AUC of the preceding 20 min as a single data point (Figure 3A). While the MD measurements had a much higher spread, in line with findings on Figure 2E, the two methods exhibited a significant linear correlation in time in response to amphetamine after the down-sampling (Figure 3B). Importantly, the two techniques positively correlated in all individual mice (Figure 3C), although not with statistical significance in each (Figure S2A).

While FP only records arbitrary fluorescence units, dialysate produced via MD and analyzed using HPLC or MS produces data that can be translated into molar values using a standard curve. Given the techniques correlate well, we aimed to use information from MD to estimate fold over baseline-values for the FP signal. We used the fit from Figure 3B to convert the z-scored FP signal to fold over baseline (Figure 3D). Zooming in on the FP trace preinjection after applying this fit (Figure 3E), we found the signal fluctuated somewhere between 0 and 4 times above the MD-estimated baseline, often reaching values close to zero. The probability density function of the signal before amphetamine injection is shown in Figure 3F. All in all, FP data are very similar to MD data when analyzed groupwise and at the same time resolution. Furthermore, *in vitro* recovery allows for estimating the absolute concentration of the extracellular space surrounding the probe (Figure S1D). After this scaling the concentration values of the FP signal fluctuated between 0 and 40 nM, with occasional peaks at higher levels (Figure S2B–D). The amphetamine-induced response had a mean concentration peak at 118.8 nM ± 7.2.

### No Information about MD Baseline in Rapid FP Signal

MD baseline values (i.e., before injection) vary both across and within subjects. However, fiber photometry only provides a relative measure, not direct information about baseline levels. We hypothesized baseline differences observed in MD might be the result of differential rapid dynamics, such as the frequency or magnitude of DA transients (Figure S3A), occluded by the lower temporal resolution of MD. To probe for shared information between the two methods, we correlated baseline concentration across mice with the relative energy across frequency bands of the FP signal (Figure 3G). No individual band survived multiple testing, but we observed a trend for fluctuations in the FP signal matching conventional DA transients (0.05–1 Hz). To further test if this domain in unison significantly correlated with baseline levels, we pooled the energy of the frequency bands and compared to MD-recorded baseline levels across mice, but found no statistical significance between the two (Figure S3B). Next, we wanted to gauge if differential fluctuations across frequencies could explain the observed intra-mouse variation in MD during baseline (Figure 3H). No significant frequency bands were identified, and the putative transient domain lost any correlation to the baseline when compared to the variation coefficient (Figure S3C).

Based on these data, we conclude that the two techniques do not share immediately apparent information at baseline, due to either high variance of MD or lack of information in the FP signal.

### Rapid Dynamics at Baseline Correlate with Magnitude of Amphetamine Response

**Figure 4:**
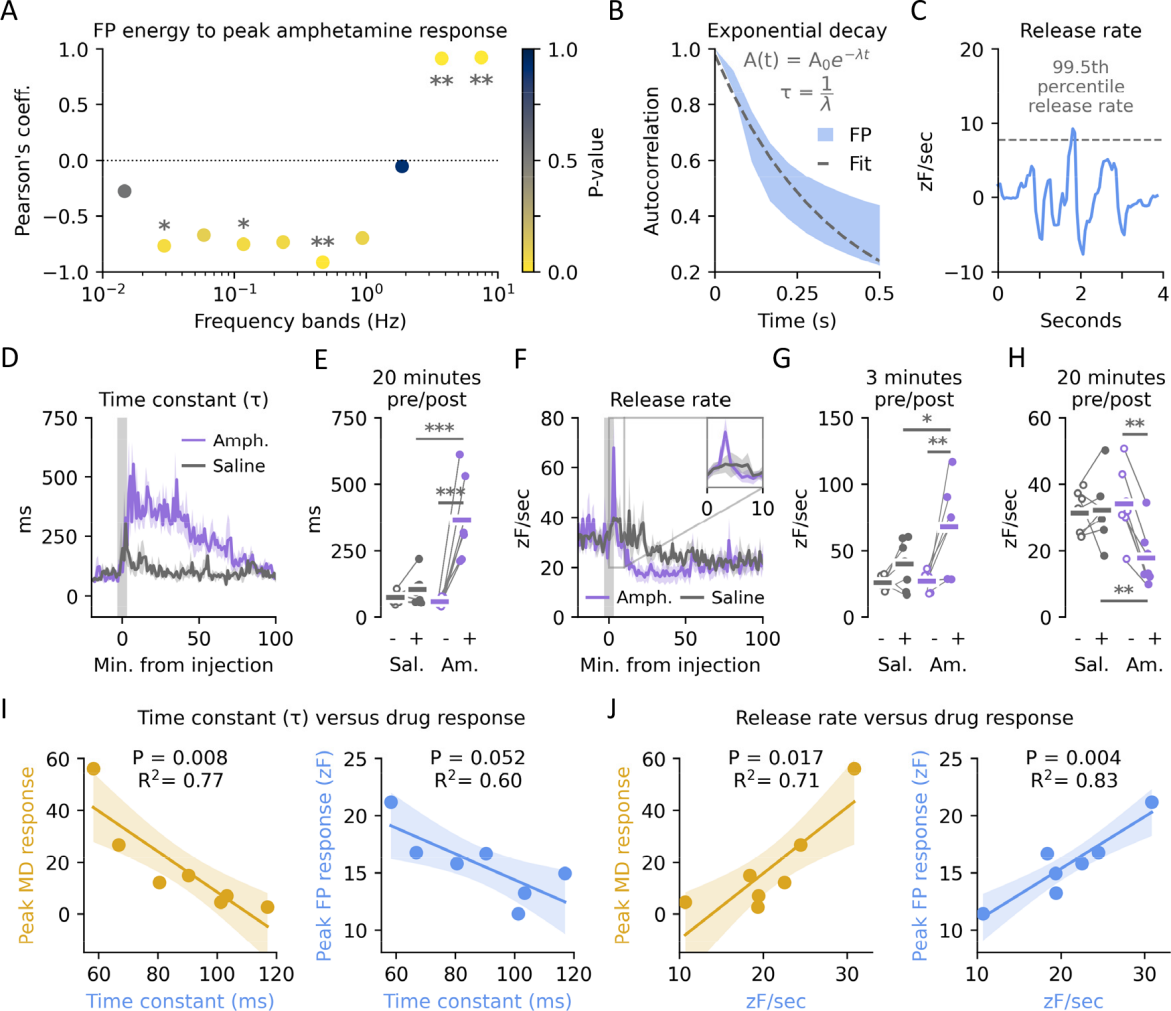
Rapid dopamine dynamics and correlation to drug effects. (A) Pearson’s coefficient between peak amphetamine response in MD and relative energy at different frequency bands in FP traces. Color indicates statistical significance. Two-sided *t* test corrected for multiple testing with Bonferroni, *n* = 7 per test, (*) *p* < 0.05, (**) *p* < 0.01. (B) Schematic of the quantification of the time constant (τ). An exponential decay is fitted to the autocorrelation and τ derived from the inverse of λ. Shaded area indicates SEM of autocorrelation for all traces preinjection and dashed line a representative exponential decay fit. (C) Representative first order derivative trace. Release rate is set to the 99.5th percentile. (D) Time constant (τ) in 1 min bins for amphetamine and saline-injected mice before and after injection. Shaded areas indicate SEM. (E) Time constant (τ) 20 min before and after injection with either saline (Sal) or amphetamine (Am). One-sided two-way *t* test corrected for multiple testing with Bonferroni, *n* = 7 per group, saline:pre, *p* = 0.17; pre:pre, *p* = 1.00; amph:pre, *p* = 0.001; amph:saline, *p* = 0.0003. (F) Release rate in 1 min bins for amphetamine and saline-injected mice before and after injection. Inset shows zoom of the first 10 min. Shaded areas indicate SEM. (G) Release rate 3 min before and after injection with either saline or amphetamine. One-sided two-way *t* test corrected for multiple testing with Bonferroni, *n* = 7 per group, saline:pre, *p* = 0.47; pre:pre, *p* = 1.00; amph:pre, *p* = 0.028; amph:saline, *p* = 0.034. (H) Release rate 20 min before and after injection with either saline or amphetamine. Two-sided two-way *t* test corrected for multiple testing with Bonferroni, *n* = 7 per group, saline:pre, *p* = 0.42; pre:pre, *p* = 0.28; amph:pre, *p* = 0.008; amph:saline, *p* = 0.01. (I) Linear correlation between time constant and peak MD or FP amphetamine response. MD: *P* = 0.008, *R*^2^ = 0.77, *n* = 7. FP: *P* = 0.052, *R*^2^ = 0.60, *n* = 7. (J) Linear correlation between release rate and peak MD or FP amphetamine response. MD: *P* = 0.017, *R*^2^ = 0.71, *n* = 7; FP: *P* = 0.004, *R*^2^ = 0.83, *n* = 7.

A strength of biosensors is their potential ability to measure rapid dynamics at sampling rates even beyond conventional FSCV.^9^ Several studies have shown how these fluctuations encode behavior.^24−26^ In our case, we wanted to investigate whether rapid dynamics at baseline could have predictive power for future drug response. Mimicking Figure 3G,H, we correlated relative energy across frequency bands of the FP signal to peak MD amphetamine response (Figure 4A). We found statistical significance of a negative correlation in several of the slower domains (<1 Hz), whereas the most rapid domains correlated positively. We hypothesized these rapid correlates were proxies for uptake and release capacity. And as amphetamine is suggested to block DAT recycling of extracellular DA, induce DA release from dopaminergic terminals, and promote DA efflux through DAT,^14,15^ we further hypothesized measures of uptake and release would predict amphetamine response potency. To that end, we construed two parameterless measures: the time constant (τ) of the decay in the autocorrelation as an indicator of DAT capacity (Figure 4B), and the 99.5th percentile of the derivate as an indicator of maximal release rate (Figure 4C). We avoided threshold-based peak detection methods, as we have previously reported dopamine dynamics in the dorsal striatum to be continuous^26^ and because these analyses require user defined parameters that may skew the results obtained.

Consistent with the ability of amphetamine to block reuptake of extracellular DA through DAT, we found a strong effect on τ after amphetamine injection (Figure 4D). Prior to administration we found a τ of approximately 100 ms (Figure 4E), consistent with previously reported values from FP measurements in DS.^9,27^ Within 5–10 min of administration τ rose to 300–400 ms, whereas saline-injected mice only showed a transient response to injection (Figure 4E). Likewise, the 99.5th percentile of the derivate, as a proxy for release rate, was affected by amphetamine (Figure 4F). This effect peaked significantly higher than the saline response after just a few minutes (Figure 4G) but quickly subsided and then undershot the saline injected mice for close to an hour (Figure 4H).

To test if these two parameters could be used in future experiments to predict drug response, we correlated the preadministration values to the individual amphetamine responses. As the FP traces are z-scored, careful consideration should be exercised, but our MD measurements are free from this caveat. For DAT capacity as a predictor, τ negatively correlated with peak amphetamine response in MD but fell short of statistical significance when compared to the FP readout, despite a similar trend (Figure 4I). This negative correlation might be explained by a higher expression of DAT leading to a less efficient inhibition at the subsaturating concentrations we administered amphetamine at. Conversely, the predrug release rate positively correlated with the subsequent amphetamine response (Figure 4J). This correlation was statistically significant for both the MD and FP response and could reflect a higher propensity for release, by either excess loading of vesicles, release probability, or number of vesicles docked.

In summary, amphetamine affected the rapid DA fluctuations as observed with FP, and both uptake and release parameters of the signal preinjection correlated with peak amphetamine response as measured by both MD and FP.

## Discussion

Robust measurements of extracellular neurotransmitter concentrations are imperative to improve our understanding of brain function and disease states. These have previously been performed with methods such as MD and FSCV, but they have limitations with either temporal resolution, neurochemical specificity, or maximal duration of recording. This limits their usefulness in longitudinal within-animal studies necessary to understanding disease progression and treatment. Recently, a new methodological paradigm has been opened by genetically encoded biosensors in combination with optical imaging technologies. When combined, these have potential to capture rapid dynamics over extended periods of time and an expanding palette of tools and techniques now allow for advances, such as multichannel recordings and depth resolution.^28,29^ The validity of the rapid signals has already been substantiated through comparisons to FSCV^9^ and enables extensive studies of behaviorally related, fast neurochemical changes.^26,30^ A thorough validation and comparison to established methods designed to monitor neurotransmission over longer periods must also be performed.

To contribute to this, we directly compared FP recordings of the DA sensor dLight1.3b to MD, as this technique has been the gold standard for assessing neurotransmitter dynamics over time scales of many minutes to hours. We did this by performing concurrent, within-mice measurements of extracellular DA in the dorsal striatum, before and after exposure to amphetamine or saline. While the two measures correlated well on a minutes-to-hours time scale after down-sampling of FP, several parameters of interest could be extracted via the biosensor that were not achievable with MD. Importantly, the rapid dynamics observed have previously been shown to correlate well with FSCV for most applications.^9^ Additionally, FP showed an almost two orders of magnitude lower variation. One potential factor is the experimental differences of the implants. The MD probe is inserted acutely into the brain parenchyma on day of experiment, whereas the lens for FP is inserted during surgery and does not generate an additional acute lesion at the time of experiment. Further, photometry samples light from the presumably less injured tissue beneath the implant site. Overall the data suggest fewer animals would be needed for a significant effect size if FP is used in place of MD.

As *in vitro* recovery allows us to assess absolute concentrations in MD, we utilized the strong fit between MD and FP to estimate DA concentrations in the biosensor-based photometry measurements. This put the peaks of spontaneous DA transients somewhere between 30 and 60 nM, which fits well with previous reports from FSCV.^31^ It is worth noting that we did not perform no-net-flux or modelled tortuosity or a trauma layer.^32^

As we expressed the sensor under a pan-neuronal promoter, we assume the FP data reflect mean concentration in the extracellular space below the fiber and do not rule out the possibility that local concentrations in microdomains may be much higher. Additionally, microdialysis and FSCV require diffusion to the probe, whereas the biosensor signal may stem directly from fluorescence generated by high concentrations at the immediate site of release. However, as the diffusion speed of dopamine is high compared to current biosensor and endogenous receptor kinetics, the signal we report is likely not confined to compartmentalized synapses.^33^ Further, dopamine in the striatum is often assumed to maintain tonic levels.^16^ We find no steady levels in our experiments on freely moving, awake animals, where dopamine fluctuates rapidly from local maxima to troughs close to the detection limit.

We also wanted to investigate if rapid dynamics at baseline could predict a future drug response. Post-hoc analysis of baseline parameters showed release rate positively correlated with maximal DA response to amphetamine as measured by both MD and FP, whereas transient decay positively correlated with amphetamine response in MD. One reason decay may not significantly correlate with FP amphetamine response is the z-scoring of the signal. Wide transients lead to a more compressed signal after normalization, which affects the subsequent amphetamine response readout.

We believe that a predictive analysis of baseline DA fluctuations in dorsal striatum could enable a preselection of stratified animal groups for studies investigating pharmacokinetic or pharmacodynamic properties of compounds in a drug discovery setting. For example, behavioral and cognitive neuroscience often splits animals into high and low performers.^34,35^ Basal dynamics may also help selecting experimentally interesting cohorts in the future in a similar manner. Finally, provided future improvement in temporal resolution of neurotransmitter measurements in humans, these analytics might extend into a preselection of subgroups within a disease^36^ to design more selective and effective clinical trials.

## Methods and Materials

### Surgery

Male C57Bl/6J mice (3–5 months old, Taconic or Janvier) were used. Mice were housed under a 12 h light/dark cycle under controlled conditions for regular indoor temperature (21 ± 2 °C) and humidity (55 ± 5%) with food and tap water available ad libitum.

Mice were anesthetized with isoflurane (5% induction, 1.5–2% maintenance in O_2_/N_2_O) and placed in a stereotaxic apparatus with mouse adaptor for teeth and ears for head fixation (Kopf 1900). 0.1 mL of Marcain (2.5 mg/kg) was injected under the shaven scalp for local analgesia before incision. The exposed skull was dried with a sterile swab. Three holes were drilled: one for anchor screw, one for the MD guide cannula (Charles River), and one for the 200 μm fiber optic lens (Neurophotometrics). The screw was inserted, and AAV9/2-hSyn1-dLight1.3b was injected into the dorsal striatum using a programmable infusion pump (Drummond Nanoject II, 5 nL/s) (virus injection coordinates: +1.18 mm anterior to bregma (AP), ±1.7 mm laterally (ML), alternating between left and right hemispheres between animals, −2.8 (100 nL), −3.0 (300 nL), and −3.2 mm (100 nL) ventral to dura (DV)). Next, the guide cannula was lowered to the following stereotaxic coordinates: +1.18 mm AP, ±1.7 mm ML, −2.8 DV. Next, dental cement (PHYMEP Superbond) was used to secure the guide cannula in place, making sure not to obstruct the bore hole of the other hemisphere, leaving it clear for implantation. After drying, the FP lens was implanted in the opposite hemisphere, in the same bore hole the virus was injected: +1.18 AP, ±1.7 ML, −3.0 DV. The cement cap was expanded to the other hemisphere, cementing the two implants into a single, very stable implant. The surgery was finalized by placing the skin over the base of the implant, secured using a single stitch. After surgery the mouse was placed in a cage under a heat lamp to wake up and then single housed. The animals received antibiotics (Noromox, 150 mg/mL amoxicillin sc) and analgesics (Norodyl, 0.25 pellet containing 2 mg/pellet Carprofen twice daily) for 5 days following surgery. Following a period of 4–12 weeks for biosensor expression a combined MD and FP study was performed.

### *In Vivo* Microdialysis and Fiber Photometry Recordings

On the day of the experiment an MD probe (0.3 mm diameter, 1 mm length, Charles River) was inserted through the guide cannula and a fiber optic cable (Doric Lenses) was attached to the lens using a ceramic mating sleeve (Doric Lenses). The microdialysis probe was perfused with filtered Ringer solution (145 mm NaCl, 3 mM KCl, 1 mM MgCl_2_, 1.2 mM CaCl_2_; 1 μL/min) throughout the study. The probe was connected to the microdialysis pump (CMA 400) and a fraction collector (810 microsampler, Univentor). The perfusion buffer consists of artificial CSF, and the probe is perfused throughout the experiment. Dialysis setup allows the animal to move freely during the trial in a bowl containing a layer of bedding. Experimental design consisted of a collection of brain dialysate samples in 20 min fractions. Prior to the first sample collection the probes had been perfused for 180 min. A total of 9 fractions were sampled (4 basal fractions and 5 postinjection fractions), and dialysate DA content was analyzed using HPLC detection. At the same time, a fiber photometry signal was recorded at 20 Hz through a fiber optic cable connected to a FP3001 (Neurophotometrics). Mice were injected with saline or amphetamine (1.5 mg/kg) sc. After the experiments animals were sacrificed and the brains removed and stored for probe placement verification.

### *In Vitro* Recovery

Five unused MD probes of the same type (0.3 mm diameter, 1 mm length, Charles River) were lowered into Eppendorf tubes containing a solution of 120 nM DA in aCSF. We collected triplicate samples per probe of 20 min each at 1 μL/min flow. Concentration of DA in dialysates was determined by means of HPLC with electrochemical detection, and *in vitro* recovery of DA was calculated.

### HPLC

Concentration of DA in dialysates was determined by means of HPLC with electrochemical detection. Samples were stored refrigerated in a CMA/200 microinjector, and separation was performed by reverse phase liquid chromatography (ODS 150 mm × 3.2 mm column) using mobile phase (150 mM NaH_2_PO_4_, 4.8 mM citric acid monohydrate, 3 mM dodecyl sulfate, 50 μM EDTA, 8 mM NaCl, 11.3% methanol, and 16.7% acetonitrile, pH 5.6) at a pump flow rate of 0.4 mL/min. Electrochemical detection was accomplished using a coulometric detector and a SenCell (Antec); potential was set at E1 = 500 mV (Coulochem III, ESA). Limit of detection was 5 fmol/20 μL.

### Fiber Photometry Preprocessing

Preprocessing of the fluorescence traces was done by applying a 5 s median filter to both the 415 and 470 nm channel from the start of recording to 2 min before the first injection. A linear fit between fluorescence intensities of the two channels was obtained and applied to the isosbestic 415 nm channel to correct for differential bleaching rates. This fit was applied to the entire 415 nm time series, and the two signals were converted to d*F*/*F* by subtracting the 415 nm channel from the 470 nm and dividing by the 415 nm. Fluctuations between 10 and 20 Hz were filtered due to lack of resolution, as per the Nyquist criterion, by applying a discrete wavelet transform. Lastly, each trace was z-scored to the mean and standard deviation of the 40 min leading up to injection.

### Fiber Photometry Analysis

Slow fluctuations below 0.01 Hz were extracted by applying the discrete wavelet transform with the “sym4” wavelet using the python PyWavelets v1.2.0 package, and for the kinetic analysis peaks were found using the python SciPy v1.5.2 package. To compare MD and FP, we computed area under the curve of FP signal in 20 min bins leading up each MD sampling and divided by time. A linear fit was obtained between all MD and transformed FP samples across mice and was uniformly applied to all transformed FP traces to convert into fold over baseline. For the baseline comparison energy was computed by summing the squares of the energy across levels and comparing the energy in each level across mice with either the mean basal levels or variance of the basal levels.

### Statistics

The employed statistical analyses are presented in the legends associated with each figure, and multiple testing was corrected for using the Bonferroni–Holmes method where specified. All *n*-values are individual mice unless otherwise specified. Statistical analyses were carried out with the open-source python packages SciPy v1.5.2, NumPy v1.18.1, and Seaborn v0.11.0. Boxplots show 25th and 75th percentile, with whiskers indicating data up to 1.5 times the interquartile range. Remaining data are plotted as outliers. No statistical methods were used to predetermine sample sizes. Data analysis was not performed blind but were automated to a degree where the experimenter had no specific impact on outcome. Sessions with severe fiber tangling were excluded from analysis on a qualitative basis, and MD samples with inconclusive peak detection were excluded.

## Notes

**Data and Code Availabity.** Raw fiber photometry and microdialysis data are available at GitHub alongside code to preprocess the data as described in the manuscript (https://github.com/Ejdrup/microdialysis-photometry-comparison). Additional data requests are available from the corresponding author upon reasonable request.
