# Supplementary material for: Within-Mice Comparison of Microdialysis and Fiber Photometry-Recorded Dopamine Biosensor during Amphetamine Response
Source: ACS Chem Neurosci. 2023 Apr 12;14(9):1622–30. doi: 10.1021/acschemneuro.2c00817 (PMC10161225; doi:10.1021/acschemneuro.2c00817)
Supplement: Supplementary file 1 — cn2c00817_si_001.pdf [file cn2c00817_si_001.pdf]

Supporting information

Within-mice comparison of microdialysis and fiber photometry-recorded dopamine biosensor during amphetamine response

Aske L. Ejdrup[1,3,*], Joel Wellbourne-Wood[1,*], Jakob K. Dreyer[2], Nina Guldhammer[1], Matthew D. Lycas[3], Ulrik Gether[3], Benjamin J. Hall[1], Gunnar Sørensen[1,**]

[1]Department of Circuit Biology, H Lundbeck A/S, Valby, 2500, Denmark

[2]Department of Bioinformatics, H Lundbeck A/S, Valby, 2500, Denmark

[3]Department of Neuroscience, Faculty of Health and Medical Sciences, Maersk Tower 7.5, University of Copenhagen, Copenhagen, 2200, Denmark

*These authors contributed equally to the work

**Correspondence: gusq@lundbeck.com

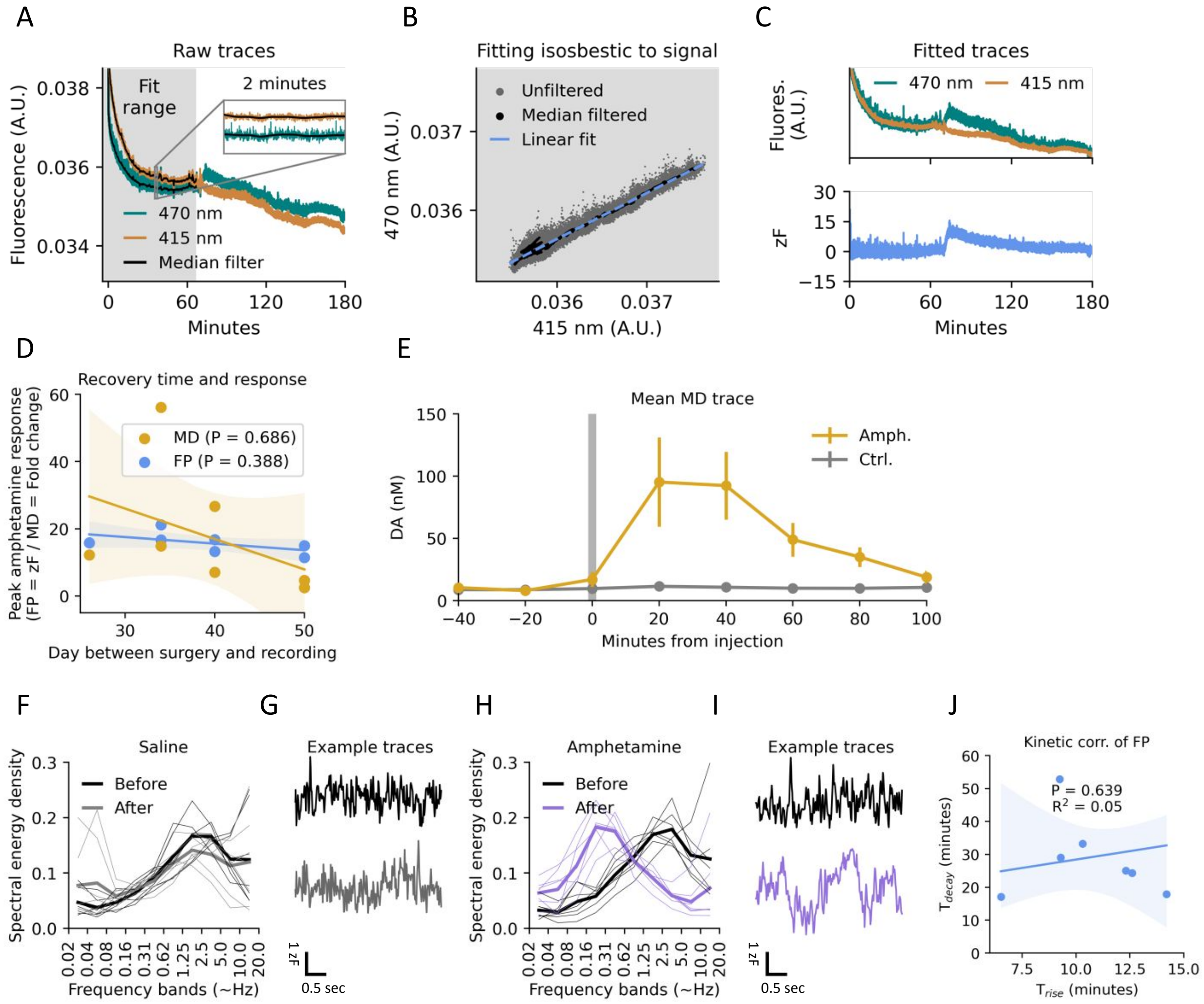

Figure S1

(A) Representative traces of both isosbestic (415 nm) and excitatory (470 nm) channels. Signal is corrected for photobleaching and z-scored from a fit in the grey area.

(B) Linear fit between the isosbestic (415 nm) and excitatory (470 nm) channel used to correct for photobleaching.

(C) Top: Representative traces after the isosbestic (415 nm) channel has been fitted to the excitatory (470 nm). Bottom: Final signal after subtraction of fitted isosbestic channel (415 nm) and z-scoring.

(D) Correlation between recovery days from surgery to recording and amphetamine response. Neither MD ($P = 0.686$, $R^2 = 0.18$, $n = 7$) nor FP ($P = 0.388$, $R^2 = 0.31$, $n = 7$) shows

any statistically significant linear correlation between the two variables. Note that the maximal wait time for the amphetamine cohort was 50 days. Shaded area indicated 95% C.I. of the linear fit.

(E) Mean MD traces from Figure 2B converted to DA concentrations after *in vitro* recovery at 8.3 % ± 1.1.

(F) Spectral energy density before and after saline injection. Thick lines indicate mean of group.

(G) Representative traces of rapid dynamics before and after saline injection.

(H) Spectral energy density before and after amphetamine injection. Thick lines indicate mean of group.

(I) Representative traces of rapid dynamics before and after amphetamine injection.

(J) No significant linear correlation observed between rise time and decay of amphetamine response measured by FP representing dLight1.3b fluorescence ($P = 0.639$, $R^2 = 0.047$, $n = 7$). Shaded area indicates 95% C.I.

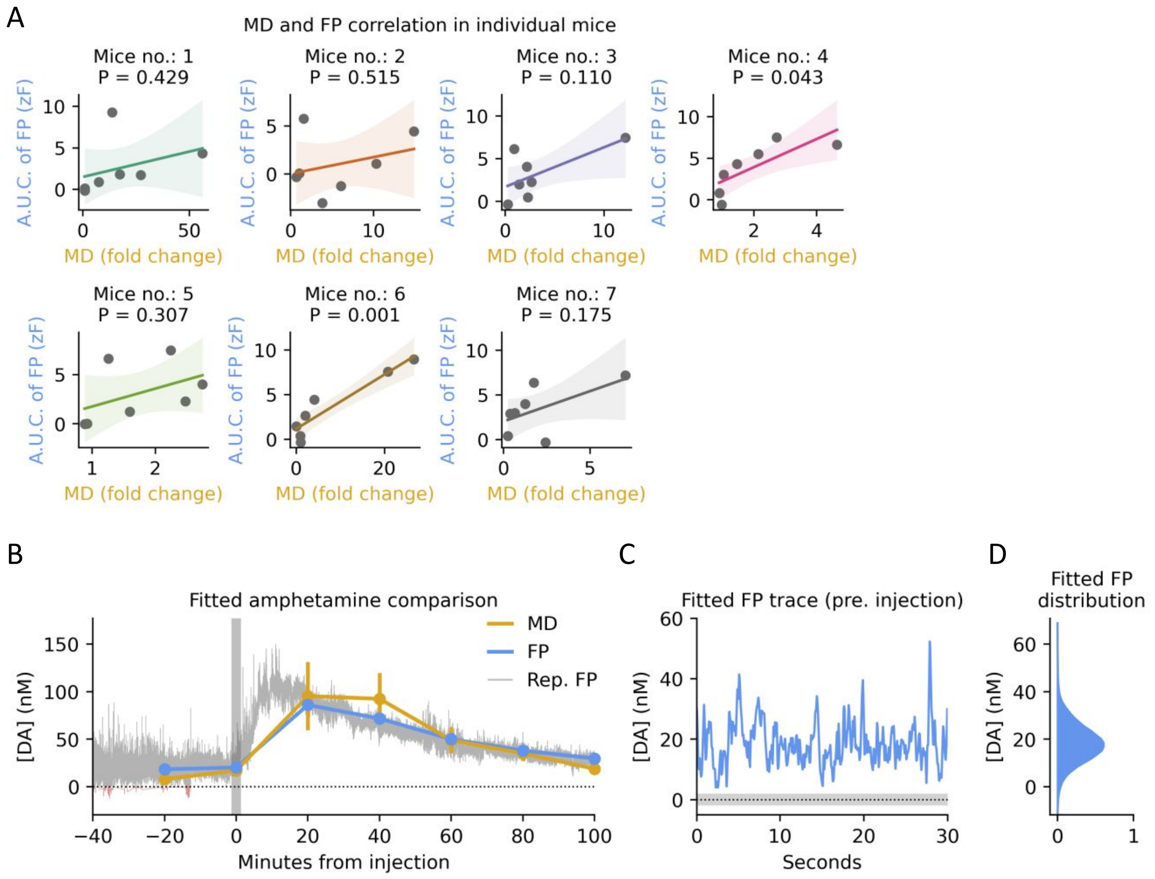

Figure S2

(A) Linear correlation between MD and down sampled FP for each amphetamine-injected mouse. Colours match traces from Figure 2A, C.

(B) Mean of down sampled FP during amphetamine injection fitted to MD by linear regression as in Figure 3D converted to DA concentration after *in vitro* recovery at 8.3 % ± 1.1. Background trace is a representative, fitted, unfiltered FP trace. Error bars indicate S.E.M.

(C) Representative fitted FP trace before injection as in Figure 3E converted to DA concentration after *in vitro* recovery. Shaded area indicates 95% C.I. of intercept in (B).

(D) Probability density function (PDF) of FP values across all mice after application of fit in Figure 3F converted to DA concentration after *in vitro* recovery. Only pre-injection data are included.

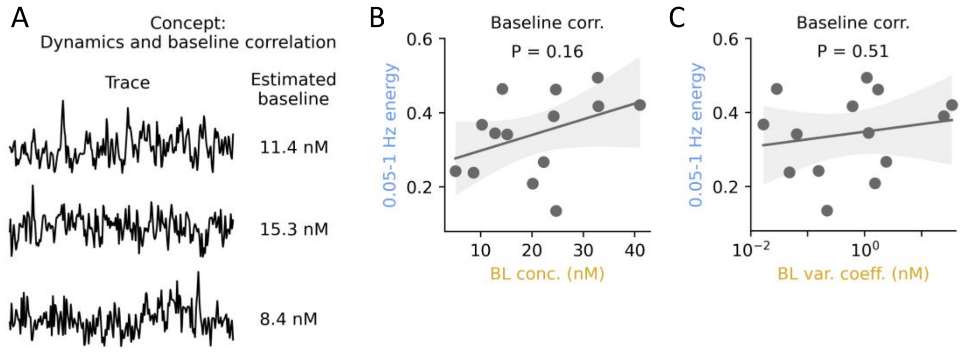

Figure S3

(A) Conceptual schematic of the hypothesis: some attributes in the rapid dynamics of the traces (left) can be used to predict the MD-measured baseline concentration (right).

(B) Correlation between MD baseline concentration and energy in putative transient frequency domain. Shaded area indicates 95% C.I. Linear regression, P = 0.16, n = 14.

(C) Correlation between MD baseline variation and energy in putative transient frequency domain. Shaded area indicates 95% C.I. Linear regression, P = 0.51, n = 14.
